# Lipids and Atherogenic Indices Fluctuation in Rheumatoid Arthritis Patients on Long-Term Tocilizumab Treatment

**DOI:** 10.1155/2018/2453265

**Published:** 2018-10-14

**Authors:** Fabio Cacciapaglia, Maria Grazia Anelli, Angela Rinaldi, Marco Fornaro, Giuseppe Lopalco, Crescenzio Scioscia, Giovanni Lapadula, Florenzo Iannone

**Affiliations:** Rheumatology Unit–Department of Emergence Medicine and Transplantation (DETO), University of Bari “Aldo Moro”, 70124 Bari, Italy

## Abstract

Rheumatoid arthritis (RA) patients are at high risk of cardiovascular (CV) events, and the chronic inflammatory state may generate quantitative and qualitative changes in lipoprotein fractions. The anti-IL-6 receptor tocilizumab (TCZ), even if effective in inflammation and joint damage prevention, determined significant alterations to RA patients' lipid levels in randomized controlled trials, but real-world data are lacking. We evaluated the changes in lipid fraction levels and disease activity in a longitudinal cohort of RA patients on long-term treatment with tocilizumab (TCZ) in a community setting. We retrospectively selected 40 naïve-biologic RA patients on treatment with intravenous TCZ compared to 20 RA patients on methotrexate treatment as the control group. Total cholesterol (Tot-Chol), low-density lipoproteins (LDL), high-density lipoprotein (HDL), and triglyceride (TG) levels were measured at the baseline and at 12, 24, and 52 weeks thereafter. At the same points, 28-joint disease activity score (DAS28), clinical disease activity index (CDAI), and EULAR clinical responses were also assessed. During the first 24 weeks, we observed in TCZ-treated patients a progressive statistically significant (*p* < 0.001) increase in Tot-Chol, LDL, HDL, and TG, which returned close to the baseline at 52 weeks. But no changes in the lipid-related CV risk indices Tot-Chol/HDL and LDL/HDL ratios and the atherogenic index (log_10_ TG/HDL) were detectable. Notably, we observed a statistically significant negative correlation between changes in lipid fractions and DAS28 or CDAI. The prolonged treatment with TCZ was associated to a transient increase in cholesterol's fractions during the first 6 months of treatment, with inverse correlation to disease activity, but with no impact on surrogate lipid indices of atherogenic risk. These findings may aid clinicians in interpreting the RA patient's lipid profile in daily clinical practice.

## 1. Introduction

Patients with rheumatoid arthritis (RA) have a twofold increased risk of morbidity and mortality for cardiovascular (CV) disease [1, 2], and “traditional” CV risk factors such as high blood pressure, diabetes, smoking, and dyslipidemia are not enough to justify this risk [3–6]. According to recent evidence, the state of low-grade systemic inflammation seems to be a key element in determining a condition of “accelerated” atherosclerosis [7, 8]. Moreover, the chronic inflammation seems to be able to interfere with traditional CV risk factors such as lipoprotein metabolism, leading to quantitative and qualitative changes in triglycerides and low-density lipoprotein (LDL) and high-density lipoprotein (HDL) cholesterol fractions [5, 9, 10]. Indeed, a condition of “reverse epidemiology” has been also taken into account for RA patients [11]. RA patients with active disease, despite the low levels of total and LDL cholesterol, have increased CV risk that subsides in those patients in clinical disease remission but with a lipid profile otherwise considered “proatherogenic,” thus leading to a “lipid paradox” [5, 11]. Several studies showed a reduction in CV risk during effective control of disease activity both under synthetic and biological disease-modifying antirheumatic drugs (DMARDs) [12–15]. The antagonism of interleukin-6 (IL-6) with tocilizumab (TCZ), an IL-6 receptor antagonist, has been associated in randomized clinical trials with changes in the lipoprotein profile, with reports of significant increase in total and LDL cholesterol levels after 8–12 weeks of treatment [16, 17]. These findings can raise concern in clinical CV risk management of RA patients treated with TCZ [18], although mechanisms and long-term outcomes are still unclear [19–21]. Therefore, the aim of this study was to evaluate in a community setting the effect of one-year TCZ treatment on RA patient's lipid metabolism.

## 2. Materials and Methods

Forty patients with diagnosis of RA, according to the 2010 EULAR/ACR classification criteria [22], with an inadequate response to previous synthetic DMARD treatment and naïve to biologic agents, which had started combination treatment with intravenous TCZ (8 mg/kg every 28 days) in our outpatient clinic from the 1^st^ January until the 31^st^ December 2015, were retrospectively evaluated. Twenty RA patients treated for at least 52 weeks with methotrexate (MTX) were considered as the control group. Demographics of enrolled patients are reported in Table 1. According to daily clinical practice, all patients have been screened for latent TB infection, exposure to B and C hepatitis, HIV infection, and anamnestic exposure to the varicella zoster virus and evaluated for previous tumors. Subjects treated with steroid > 7.5 mg/day prednisone equivalent, lipid-lowering agents, and/or smoking more than 10 cigarettes per day were excluded from the study. The functional capacity of patients was evaluated with the Health Assessment Questionnaire Disability Index (HAQ-DI).

At the baseline and after 12, 24, and 52 weeks of treatment, the DAS28-ESR and CDAI were saved, and the treatment response according to the EULAR definitions [23] was assessed. At the same time, plasma levels of total cholesterol (Tot-Chol), low-density lipoproteins (LDL), high-density lipoprotein (HDL), and triglycerides (TG), added to the routine rheumatology laboratory tests and assessed according to standard laboratory protocol, were retrieved. The non-HDL-cholesterol (Tot-Chol-HDL) levels, the lipid-related CV risk indices Tot-Chol/HDL and LDL/HDL ratios, and the atherogenic index of plasma (AIP: log_10_ TG/HDL) were also calculated [24–26].

As part of the GISEA registry protocol approved by the local Ethical Committee (Trial.Gov NCT01543594), all patients had signed a written informed consent before starting the treatment, and this observational study was conforming to the ethical guidelines of the 1975 Declaration of Helsinki.

### 2.1. Statistical Analysis

All data have been tested for the *D'Agostino-Pearson omnibus normality test*, and Gaussian distribution was detected; accordingly, we expressed data as mean ± standard deviation (SD) or percentage with confidence interval when appropriate. Consequently, differences for continuous variables were evaluated using the two-tailed variable analysis of variance followed by paired *t*-test, while categorical data were calculated using Fisher's probability test or the *χ*^2^-test, when appropriate. To test whether the difference in means between the groups was statistically significant, we performed the analysis of variance (ANOVA). Correlations between continuous variables were performed using the Pearson test.

The analysis of covariance (ANCOVA) was used to determine confounding effect as well as to expose eventual interaction effect of suitable variables on the relationship between lipid changes at 24 and 52 weeks of treatment with TCZ or MTX. Parameters tested were disease duration, gender, baseline mean ESR and CRP serum levels, mean lipid levels and fractions, baseline mean DAS28, mean CDAI, and mean HAQ. Confounders were fitted in the adjusted model along with TCZ treatment as the independent variable.

The calculated statistical power of the study, considering an alpha error level of 5% with a sample size of 40 patients and a nominal variation of 10 ± 5 mg/dl in lipid levels, was approximately 99%.

A *p* value < 0.05 or <0.2 was considered statistically significant in the univariate or multivariate model, as appropriate. The Statistical System Prism (GraphPad InStat, version 6.0—GraphPad Software, San Diego, CA, USA) and the Analysis System (SAS 9.3 Software—SAS Institute Inc., Cary, NC, USA) were used for all analysis.

## 3. Results

At baseline, all patients had high disease activity (DAS28-ESR > 5.1 with CDAI > 22), and, as shown in Table 1, a significant improvement of both parameters DAS28 and CDAI was achieved as early as 24 weeks and maintained until 52 weeks of treatment (*p* < 0.001). After 12 weeks of TCZ, 16 patients had an improvement in DAS28 ≥ 1.2 achieving a good EULAR response, 22 patients had a moderate response DAS28 improvement between 1.2 and 0.6, and 2 patients had no response (DAS28 improvement < 0.6), according to the treatment response EULAR definitions. Due to failure in clinical response, these last 2 patients dropped out of the study and were no further considered for the statistical analysis. After 24 weeks, further 8 patients achieved a good EULAR response, and at 52 weeks, a total of 33 patients (82.5%) had a good clinical response. The HAQ-DI of patients improved significantly from 1.7 ± 0.5 of the baseline to 1.2 ± 0.6, 0.8 ± 0.4, and 0.7 ± 0.5 after 12, 24, and 52 weeks of treatment, respectively (*p* < 0.001).

Methotrexate-treated patients were fewer female and presented lower disease duration compared to TCZ patients but had significant good response to treatment and continued MTX for 52 weeks as due by inclusion criteria.

**Figure 1:**
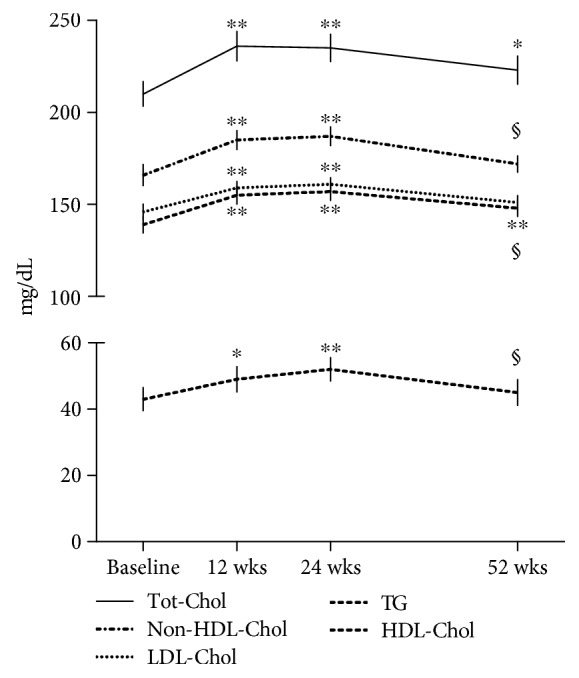
Changes in total cholesterol (Tot-Chol), non-HDL cholesterol, low-density lipoprotein (LDL) and high-density lipoprotein (HDL) cholesterol, and triglyceride (TG) plasmatic levels during tocilizumab treatment. The peak change in Tot-Chol is at 12 weeks and in non-HDL-, LDL-, and HDL-Chol, and TG is at 24 weeks; after that, lipids come down by 52 weeks but remain still significantly elevated compared to the baseline only for Tot-Chol and TG. ^∗^*p* < 0.05 and ^∗∗^*p* < 0.001 vs. baseline; §*p* < 0.05 vs. 24 weeks.

**Figure 2:**
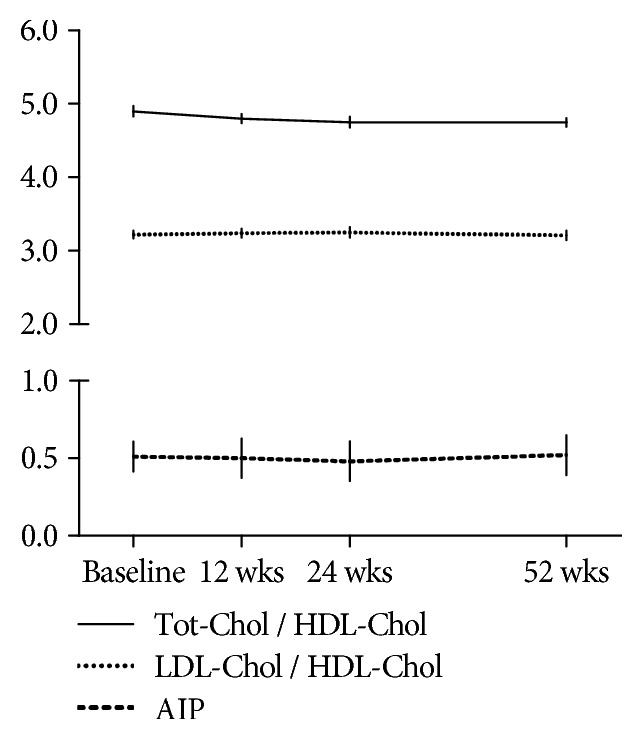
Ratios of total cholesterol/high-density lipoprotein (Tot-Chol/HDL-Chol) and low-density lipoprotein-/HDL-cholesterol (LDL-Chol/HDL-Chol) and the atherogenic index of plasma (log triglycerides/HDL-Chol; AIP) at the different time points during tocilizumab treatment. *p* = NS.

From the lipid level evaluation of enrolled patients, we detected that in the TCZ group, hypercholesterolemia (Tot-Chol > 200 mg/dl) and/or hyper-LDL-Chol levels (>130 mg/dl) at the baseline was detectable in 32 (80%) of the enrolled patients. Only 8 patients (20%) had reduced HDL-Chol levels (<40 mg/dl) and/or hyper-triglyceridemia (TG > 150 mg/dl). While in MTX-treated patients, only 3 patients (15%) had a condition of hypercholesterolemia and/or hyper-LDL-Chol levels. In Table 2 are reported the lipid fraction levels at the baseline and after 24 and 52 weeks of treatment with TCZ and MTX. After starting TCZ treatment, Tot-Chol levels had a statistically significant change already after 12 weeks with an average increase of 27 ± 13 mg/dl that reached a mean increase of 31 ± 16 mg/dl and 12 ± 15 mg/dl at 24 weeks and 52 weeks of treatment, respectively, compared to baseline levels (*p* < 0.05) (Figure 1). A similar statistically significant increase was also detected for the HDL- and LDL-Chol and TG levels, with an average increase of 12 ± 15 mg/dl, 19 ± 23 mg/dl, and 21 ± 29 mg/dl after 12 weeks, respectively, which persisted until week 24, and settle down to a mean increase of 8 ± 15 mg/dl for HDL-Chol, 6 ± 17 mg/dl for LDL-Chol, and 8 ± 24 mg/dl for TG after 52 weeks compared to baseline levels. From the evaluation of non-HDL-Chol, it was 172 ± 17 mg/dl at the baseline and increased to 191 ± 20 mg/dl and 192 ± 20 mg/dl, respectively, after 12 and 24 weeks of treatment (*p* < 0.001 vs. baseline). After 52 weeks of TCZ treatment, it returned to 179 ± 15 mg/dl (*p* = 0.07 vs. baseline; *p* < 0.01 vs. 12 and 24 weeks). Accordingly, as shown in Figure 2, the ratios Tot-Chol/HDL-Chol and LDL-Chol/HDL-Chol and the AIP remained unchanged during the period of the study, with values considered of “moderate risk,” according to the Framingham study [26–29].

In the MTX-treated patients, the lipid levels did not change significantly during treatment but were significantly lower than those observed in TCZ-treated patients (see Table 2). Similarly, the lipid ratios Tot-Chol/HDL-Chol and LDL-Chol/HDL-Chol and the AIP remained unchanged during MTX treatment but were significantly lower than those calculated in TCZ-treated patients.

From the one-way ANCOVA, we observed statistically significant differences in lipid levels after 24 and 52 weeks of treatment among TCZ and MTX arms. However, only baseline mean total cholesterol was found to confound this association, and no interaction effect was found. After adjusting for baseline variables (disease duration, gender, baseline mean ESR and CRP serum levels, baseline mean Tot-Chol, LDL- and HDL- cholesterol and TG levels, baseline mean DAS28 and mean CDAI, and mean HAQ), difference between lipid levels determined at 24 weeks among the TCZ and MTX arms persisted (*p* = 0.01), while statistical significance after 52 weeks of treatment was lost (*p* = 0.31).

**Figure 3:**
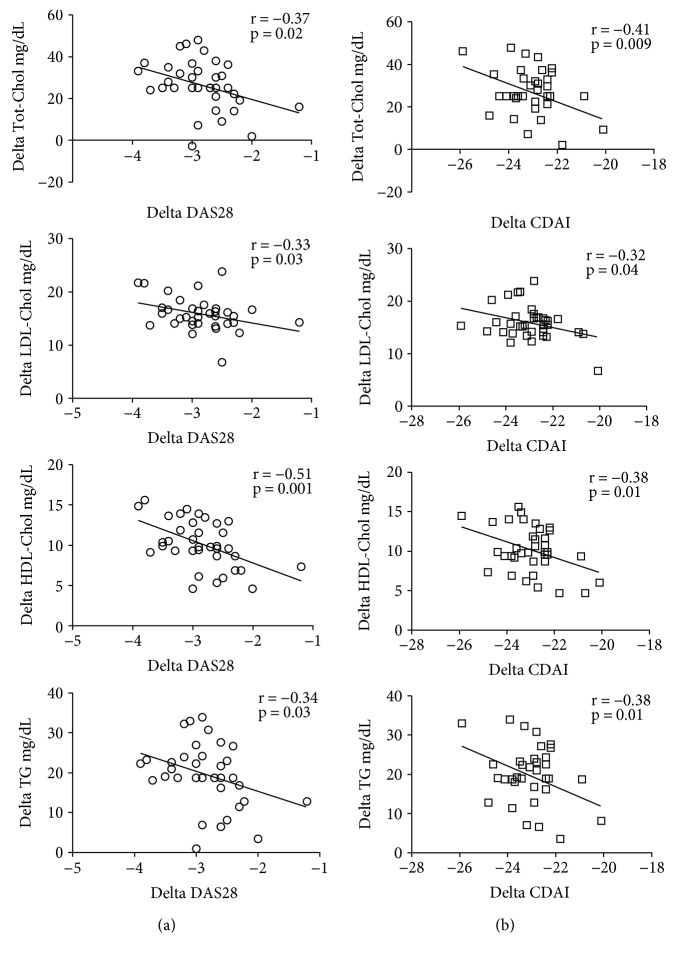
Correlations between variations (delta) in total cholesterol (Tot-Chol), low-density lipoprotein (LDL) and high-density lipoprotein (HDL) cholesterol, and triglyceride (TG) plasmatic levels and variation (delta) of the DAS28 (a) and CDAI (b) after 24 weeks of tocilizumab treatment.

Correlating the Tot-Chol, LDL- and HDL- cholesterol, and TG plasmatic level changes, with the DAS28 and CDAI changes after 24 weeks of tocilizumab (TCZ) compared to the baseline, we found a statistically significant inverse correlation between lipid levels and disease activity assessed by both scores (Figure 3). Finally, during the 1-year follow-up, there were no CV events in the studied population.

## 4. Discussion

In our observational study in a real-life setting, we confirmed that during TCZ therapy, all lipid fractions increase, but the prolonged follow-up for 12 months revealed that the increase occurs during the first 6 months of treatment, with a trend to return to baseline levels after one year.

MTX-treated patients showed no significant change in lipid fraction levels over time, confirming the key role of IL-6 in lipid metabolism. It must be highlighted that the control group with MTX had lower basal LDL cholesterol and TG levels, but this difference may be justified by different disease durations and previous csDMARDs ± steroid treatment in TCZ-treated patients.

Indeed, IL-6 exerts specific effect on hepatic protein production, such as CPR and lipoproteins, and its inhibition with TCZ had more consistent impact on CPR and lipid fractions compared to MTX-treated patients.

Previous studies with TCZ had shown an increase of Tot-Chol levels and LDL and HDL fractions [16, 17], but other studies did not show statistically significant variations [30]. However, all this information comes from randomized clinical trials and their post hoc analysis, with a follow-up of at most 24 weeks. Real-world data are missing, and only the short-term changes on lipid fractions, probably influenced by the anti-inflammatory and hepatic effects on lipid metabolism by the inhibition of IL-6, have been documented.

Hashizume et al. [31] have shown that IL-6 decreases levels of blood lipids, increasing the expression of the LDL receptor in different tissues. Moreover, Strang et al. demonstrated a significant decreased LDL receptor expression in liver cell cultures following TCZ incubation [16]. A recent study by Robertson et al. highlighted that TCZ was able to reduce LDL hypercatabolism in 11 RA patients with high disease activity, leading to Tot-Chol, LDL-Chol, and HDL-Chol elevations, without change in the Tot-Chol/HDL-Chol ratio [21]. Another study [32] reported a reduction of the secretory phospholipase A2 (sPLA2-IIA) and serum amyloid A (SAA), during therapy with TCZ, with modification in HDL lipoprotein composition. More recently, García-Gómez et al. on behalf of the CARdiovascular rheuMAtology study project reported from their database that the 30 RA patients treated with TCZ had statistically significant lower Lp(a) plasma levels compared to patients treated with nonbiologic therapies [33].

Such effects result in a potential antiatherogenic and anti-inflammatory lipoprotein's function, restoring their scavenger function to the liver. In this way, the IL-6 antagonism would result in good inflammation control despite an increase in serum lipid levels, with a favorable net effect on CV risk. The systemic inflammatory condition observed in high-disease-activity RA patients and the high IL-6 levels increases the cholesterol uptake from the liver and other tissues by the overexpression of LDL receptors [16, 31, 34]. The reduction of IL-6 levels, such as in low-disease or remission RA patients, even more during an anti-IL-6 treatment, leads to a reduction in the reuptake of cholesterol fractions from tissues with consequent increase in their circulating levels. Finally, the good disease and systemic inflammation control, obtained with prolonged effective anti-IL-6 treatment, would progressively return to a lipid metabolism regulated by the same factors of subjects without RA.

Our findings confirmed that TCZ may influence the cholesterol levels and the other lipid fractions with a comparable gain of the different lipoproteins, and we demonstrated therefore that the different lipid ratios remain unaltered. Accordingly, other authors have reported an increase, after 3 months, of all major lipoproteins during treatment with TCZ, more evident for the LDL-Chol, but with relative stability of both LDL-Chol/HDL-Chol and Tot-Chol/HDL-Chol ratios. In addition, we have demonstrated that AIP does not change during 12 months of TCZ treatment. These parameters are known to be more closely associated to the real CV risk due to lipids than the individual measures of lipid fractions, which may be mistaken by the effect of inflammation [35, 36]. To date, the AIP as a surrogate of small LDL particle size is considered a valid predictor of CV risk, especially in selected categories of patients, such as diabetics [26].

According to the inclusion criteria of our study, patients should not receive any lipid-lowering treatment, and considering the cholesterol levels achieved after 12 weeks of TCZ treatment, some patients would be eligible for such therapy. Indeed, the EULAR recommendations on CV risk management in inflammatory arthritis suggest starting statin [37], but in the case of TCZ treatment and its metabolic effects, this decision could be procrastinated after at least 6 months of drug use, also to avoid the potential muscle side effects of statin [38]. Moreover, our observation of lipid fraction increase associated with TCZ treatment should be considered “paradoxical” on CV risk, underlining once again the complex interaction between systemic inflammation, lipids, and CV risk in RA patients.

It has been demonstrated that disease activity indices were independently correlated to CV risk in RA patients, as well as a high atherogenic index at the baseline (pretreatment) may be independent of the inhibition of IL-6. Furthermore, poor control of disease activity in RA but not necessarily lipid changes, has been associated with occurrence of major adverse CV events in patients treated with TCZ [39].

Finally, the correlation of changes in disease activity and Tot-Chol, HDL, LDL, and TG levels demonstrates that the systemic inflammatory state has metabolic effects. The correlation especially between lipid fraction and CDAI, calculated without considering the CRP with hepatic secretion according to IL-6 circulating levels [40], suggests that disease activity may have a direct influence on lipid profile of RA patients.

The clear effect of these changes on the lipid-dependent CV risk should be demonstrated with long-term prospective studies without the interference of any other drugs on the lipid set.

## 5. Conclusions

Our study, with a 1-year follow-up, confirmed that TCZ may interfere with cholesterol levels; we demonstrated a more consistent effect during the first 6 months of treatment and subsequently a reverse trend to pretreatment levels.

We found a similar effect on all lipoprotein fractions; thus, no substantial differences were highlighted in the evaluated lipid ratios, Tot-Chol/HDL-Chol and LDL-Chol/HDL-Chol, and the AIP, and the net effect on lipid-related CV risk would seem irrelevant.

Further studies are needed to confirm our findings and to elucidate the intriguing mechanisms linking inflammation and lipid metabolism.

## Figures and Tables

**Table 1:** Baseline demographic characteristics of RA patients treated with tocilizumab (TCZ) and methotrexate (MTX).

	TCZ pts. (*n* = 40)	MTX pts. (*n* = 20)
Female, *n* (%)	36 (90)	12 (60)^∗^
Age (years)—mean ± SD	55 ± 11	56 ± 14
Disease duration (months)—mean ± SD	9.8 ± 4.3	6.7 ± 3.0^∗^
BMI (kg/m^2^)—mean ± SD	26.5 ± 4.2	27.2 ± 5.0
Smokers, *n* (%)	11 (27.5)	5 (25)
Arterial hypertension, *n* (%)	12 (30)	7 (35)
Diabetes, *n* (%)	6 (15)	3 (15)
RF positive, *n* (%)	32 (80)	16 (80)
ACPA positive, *n* (%)	33 (82.5)	17 (85)
Combination treatment, *n* (%)		
Steroids	14 (35)	6 (30)
csDMARD		
MTX	28 (70)	20 (100)
LFM	8 (20)	
SSZ	4 (10)	

ACPA: anticitrullinated peptide antibody; csDMARD: conventional synthetic disease-modifying antirheumatic drug; MTX: methotrexate; LFM: leflunomide; SSZ: sulfasalazine; RF: rheumatoid factor. ^∗^*p* < 0.01.

**Table 2:** Laboratory and clinical findings of RA patients treated with tocilizumab (TCZ) and methotrexate (MTX).

	TCZ pts. (*n* = 40)			MTX pts. (*n* = 20)		
(Mean ± SD)	Baseline	24 weeks	52 weeks	Baseline	24 weeks	52 weeks
ESR (mm/h)	64 ± 23	17 ± 9^∗^	15 ± 7^∗^	57 ± 23	13 ± 12^∗^	12 ± 10^∗^
CRP (mg/l)	7.3 ± 3.2	1.1 ± 3^∗^	0.9 ± 2.7^∗^	6.8 ± 1.9	3.5 ± 4^∗^§	1.7 ± 1^∗^§
DAS28	5.3 ± 0.4	2.5 ± 1.6^∗^	2.2 ± 1.1^∗^	4.9 ± 1.5	2.6 ± 1.8^∗^	1.9 ± 0.8^∗^
CDAI	28.9 ± 2.1	6.1 ± 1.1^∗^	4.7 ± 1^∗^	26.6 ± 8.6	9 ± 1.4^∗^	5.2 ± 1.1^∗^
HAQ	1.7 ± 0.5	0.8 ± 0.4^∗^	0.7 ± 0.5^∗^	1.2 ± 0.7§	0.4 ± 0.3^∗^§	0.2 ± 0.5^∗^§
Total cholesterol (mg/dl)	210 ± 21	235 ± 23^∗^†	223 ± 24^∗^	189 ± 38	188 ± 35§	192 ± 38§
Non-HDL cholesterol (mg/dl)	166 ± 18	187 ± 16^∗^†	172 ± 14	156 ± 27	146 ± 25§	142 ± 19§
LDL cholesterol (mg/dl)	146 ± 13	161 ± 11^∗^†	151 ± 12	128 ± 22§	117 ± 25§	118 ± 15§
Triglycerides (mg/dl)	139 ± 14	157 ± 15^∗^†	148 ± 14^∗^	126 ± 26§	121 ± 24§	123 ± 22§
HDL cholesterol (mg/dl)	43 ± 11	52 ± 11^∗^†	45 ± 12	50 ± 14	48 ± 12	51 ± 10
Total/HDL cholesterol ratio	4.9 ± 0.22	4.75 ± 0.23	4.75 ± 0.18	3.6 ± 0.9§	3.8 ± 0.8§	3.5 ± 0.7§
LDL/HDL cholesterol ratio	3.22 ± 0.17	3.25 ± 0.22	3.21 ± 0.2	2.2 ± 0.6§	2.5 ± 0.9§	2.1 ± 0.5§
AIP (log_10_ TG/HDL)	0.51 ± 0.3	0.48 ± 0.4	0.52 ± 0.4	0.4 ± 0.3§	0.4 ± 0.2§	0.38 ± 0.4§

AIP: atherogenic index of plasma; CRP: C-reactive protein; ESR: erytro-sedimentation rate; DAS28: 28-joint disease activity score; CDAI: clinical disease activity index; HAQ: Health Assessment Questionnaire; HDL: high-density lipoprotein; LDL: low-density lipoprotein; TG: triglycerides. Univariate analysis: ^∗^*p* < 0.001 vs. baseline; §*p* < 0.01 vs. TCZ. Multivariate analysis: †*p* = 0.01.

## Data Availability

The data about TCZ and MTX effects on lipid profile used to support the findings of this study are included within the article.
